# Effects of prehospital epinephrine administration on neurological outcomes in patients with out-of-hospital cardiac arrest

**DOI:** 10.1186/s40560-015-0094-3

**Published:** 2015-06-24

**Authors:** Yuichi Ono, Mineji Hayakawa, Takeshi Wada, Atsushi Sawamura, Satoshi Gando

**Affiliations:** Department of Emergency and Critical Care Medicine, Hokkaido University Hospital, Kita 8, Nishi 5, Kita-ku, Sapporo, Hokkaido 060-8648 Japan

**Keywords:** Out-of-hospital cardiac arrest, Resuscitation, Epinephrine, Prehospital

## Abstract

**Background:**

To determine if the effects of epinephrine administration on the outcome of out-of-hospital cardiac arrest (OHCA), patients are associated with the duration of cardiopulmonary resuscitation (CPR) performed by Emergency Medical Service (EMS) personnel.

**Methods:**

This retrospective, nonrandomized, observational analysis used the All-Japan Utstein Registry, a prospective, nationwide population-based registry of all OHCA patients transported to the hospital by EMS staff as the data source. We stratified all OHCA patients for quartile of EMSs’ CPR duration. Group 1 consisted of patients who fell under the 25th percentile of EMSs’ CPR duration (under 15 min); group 2, patients who fell into the 25th to 50th percentile (between 15 and 19 min); group 3, patients who fell into the 50th to 75th percentile (between 20 and 26 min); and group 4, patients who fell at or above the 75th percentile (over 26 min). The primary endpoint was a favorable neurological outcome 1 month after cardiac arrest. The secondary endpoints were ROSC before arrival at the hospital and 1-month survival.

**Results:**

A total of 383,811 patients aged over 18 years who had experienced OHCA between 2006 and 2010 in Japan, when stratified for quartile of EMSs’ CPR duration, the epinephrine administration increased the rate of return of spontaneous circulation (ROSC) approximately tenfold in all groups. However, the beneficial effects of epinephrine administration on 1-month survival disappeared in patients on whom EMSs’ CPR had been performed for more than 26 min, and the beneficial effects of epinephrine administration on neurological outcomes were observed only in patients on whom EMSs’ CPR had been performed between 15 and 19 min (odds ratio, 1.327, 95 % confidence intervals, 1.017–1.733 *P* = 0.037).

**Conclusions:**

Epinephrine administration is associated with an increase of ROSC and with improvement in the neurological outcome on which EMSs’ CPR duration is performed between 15 and 19 min.

## Background

The administration of epinephrine for cardiopulmonary resuscitation (CPR) has been advocated for decades [1] and is recommended in both the 2010 International Consensus on Cardiopulmonary Resuscitation and Emergency Cardiovascular Care Science [2] and the 2010 American Heart Association Guidelines for Cardiopulmonary Resuscitation and Emergency Cardiovascular Care [3]. However, a large observational before-and-after study in Singapore found that epinephrine treatment is not beneficial for immediate or 1-month survival [4]. While, in contrast, several previous studies reported that epinephrine administration increases the frequency of return of spontaneous circulation (ROSC) [5–8], they also raised doubts regarding the effects of epinephrine on the neurological outcomes in patients with out-of-hospital cardiac arrest (OHCA). Specifically, they indicated that although epinephrine administration increases the short-term survival rate in this patient population, it does not improve neurological outcomes [5–8].

The emergency medical service (EMS) in Japan is permitted to administer epinephrine to patients with OHCA. In a recent large prospective observational propensity analysis of epinephrine administration to OHCA patients using the All-Japan Utstein database, Hagihara et al. [9] concluded that prehospital epinephrine administration to patients with OHCA significantly increases the likelihood of ROSC before hospital arrival but is not associated with an increase in either survival or good functional outcome 1 month after the event. Although Hagihara et al. as well as other previous studies failed to demonstrate the effectiveness of epinephrine treatment in improving neurological outcomes in patients with OHCA, epinephrine administration during CPR has long been, and remains, an internationally accepted treatment.

In support of epinephrine treatment, we hypothesized that the poor results observed in previous studies reflect their lack of consideration of the duration of CPR performed by EMS staff before the patient’s arrival at the hospital. The interval is the period when specially trained EMS personnel are able to administer epinephrine. Specifically, we hypothesized that a shorter duration of EMSs’ CPR would reduce the likelihood of epinephrine administration and be more likely to result in favorable outcomes, whereas a longer duration of EMSs’ CPR would increase the likelihood of epinephrine administration and be more likely to result in unfavorable outcomes. To test the hypothesis that the effects of epinephrine administration on patient prognosis depend on the duration of CPR performed by EMS personnel, we evaluated the effect of the performance of EMSs’ CPR of four durations on the neurological outcomes of OHCA patients.

## Methods

### Study design

This study was a retrospective analysis of prospectively collected data contained in the All-Japan Utstein Registry of OHCA patients. The registry was initiated in January 2005 as a prospective nationwide population-based registry of all OHCA patients transported to the hospital by EMS staff and is managed by the Fire and Disaster Management Agency (FDMA). As the public has access to the data contained in the registry, the Institutional Review Board of Hokkaido University Hospital for Clinical Research waived the requirements to obtain written informed consent from the patients included in the database and to submit a study plan.

### Japanese EMS system characteristics and procedures

The Japanese EMS system has been described previously [10–12]. In most cases, the ambulance crew consists of three emergency team members. One member is specially trained in EMS, specifically the provision of prehospital emergency care, and is permitted to insert an intravenous line and advanced airway device (i.e., laryngeal mask airway, laryngeal tube, or esophageal-tracheal twin-lumen airway) and use semiautomated external defibrillators. Although this specially trained EMS team member is permitted to administer epinephrine and insert an endotracheal tube with the approval of the online emergency physician, in many regions of Japan, he or she is not permitted to administer epinephrine to OHCA patients with asystole as the primary electrocardiogram (ECG) rhythm and/or without a bystander acting as a witness.

When cardiac arrest is diagnosed, chest compression and ventilation using a bag-valve mask are immediately initiated, and CPR is provided by the EMS personnel according to international guidelines [3]. If necessary, the EMS personnel insert an advanced airway device and apply a semiautomated external defibrillator, after which they attempt to gain peripheral venous access to administer 1 mg of epinephrine intravenously every 3 to 5 min until the ROSC or arrival at the hospital. No drugs other than epinephrine are permitted for use by EMS personnel in Japan. After attempting defibrillation, inserting an advanced airway device, and administering epinephrine, the EMS staff transfer the patient to the hospital while performing CPR. If unable to gain peripheral venous access at the scene, they again attempt to gain peripheral venous access in the ambulance after departing from the scene. Upon arrival at the hospital, the patient is provided with advanced life support, including the administration of epinephrine.

### Patient selection

The patients in this study were selected from among all patients who had experienced OHCA before the arrival of EMS personnel and were subsequently treated by EMS personnel and transported to a medical institution in Japan between 1 January 2006 and 31 December 2010. We excluded patients under 18 years of age; in whom spontaneous circulation had been restored before the arrival of EMS personnel; for whom medical records were missing data; whose condition had unlikely been due to cardiac arrest; for whom more than 480 min had elapsed from the emergency call to hospital arrival, more than 60 min from the emergency call to the initiation of CPR, or more than 120 min from the initiation of CPR to hospital; who had been transferred with a physician rather than with specially trained EMS personnel; and/or whose OHCA episode had been witnessed by EMS personnel.

### Patient grouping

The patients were divided into four groups based on EMSs’ CPR duration, defined as the interval from the initiation of CPR by EMS staff to ROSC or arrival at the hospital. The interval is the period when specially trained EMS personnel are able to administer epinephrine. Group 1 consisted of patients who fell under the 25^th^ percentile of EMSs’ CPR duration (under 15 min); group 2, patients who fell into the 25^th^ to 50^th^ percentile (between 15 and 19 min); group 3, patients who fell into in the 50^th^ to 75^th^ percentile (between 20 and 26 min); and group 4, patients who fell at or above the 75^th^ percentile (over 26 min). To assess baseline patient characteristics, the patients were further divided into the epinephrine administration group and the non-epinephrine administration group.

### Data collection

The duration of all procedures was recorded with the timekeeping device used by each EMS system, which recorded receipt of the emergency call by the EMS, ambulance arrival at the scene, initial contact with the patient, initiation of CPR, and arrival at the hospital. The data collected included patient sex and age, initial cardiac rhythm, and time course of resuscitation, as well as whether a bystander had witnessed the episode of cardiac arrest and/or initiated CPR, the patient had been intubated, epinephrine had been administered, or spontaneous circulation had been restored before arrival at the hospital. One month after the event, follow-up data were collected regarding survival and neurologic status by the EMS staff person in charge of the patient with OHCA during a meeting with the medical control director at the hospital. In partnership with the medical control director, the emergency personnel summarized the data for each OHCA case in standardized Utstein style. Using these procedures, the data collected at approximately 800 fire stations maintaining dispatch centers in 47 prefectures were integrated into the national registry system on the FDMA database server.

### Outcome investigation

The primary endpoint was a favorable neurological outcome 1 month after cardiac arrest. A favorable neurological outcome was defined as a cerebral performance category score of 1 (good performance) or 2 (moderate disability) and an unfavorable neurological outcome as a score of 3 (severe cerebral disability), 4 (vegetative state), or 5 (death) [13–16]. The secondary endpoints were ROSC before arrival at the hospital and 1-month survival.

### Statistical analysis

The patient characteristics and outcomes were compared between two groups using Student’s *t*-test for numerical variables and the chi-square test for categorical variables. The adjusted odds ratios (ORs) and 95 % confidence intervals (CIs) for outcomes were assessed by performing logistic regression analysis that included the variables of age, sex, bystander eyewitness, type(s) of CPR techniques initiated by a bystander (chest compression, rescue breathing, and/or the use of a public-access automated external defibrillator [AED]), cause of cardiac arrest, primary ECG rhythm, type(s) of life support care provided by EMS personnel (defibrillation, rescue breathing, and/or advanced airway management), and duration of all events. The SPSS 15.0J statistical software package (SPSS Inc., Chicago, IL, USA) was used for all statistical analyses. A *P* value of <0.05 was considered to be statistically significant. Unless otherwise indicated, all data are expressed as the mean (percentage).

## Results

### Patient selection

**Fig. 1:**
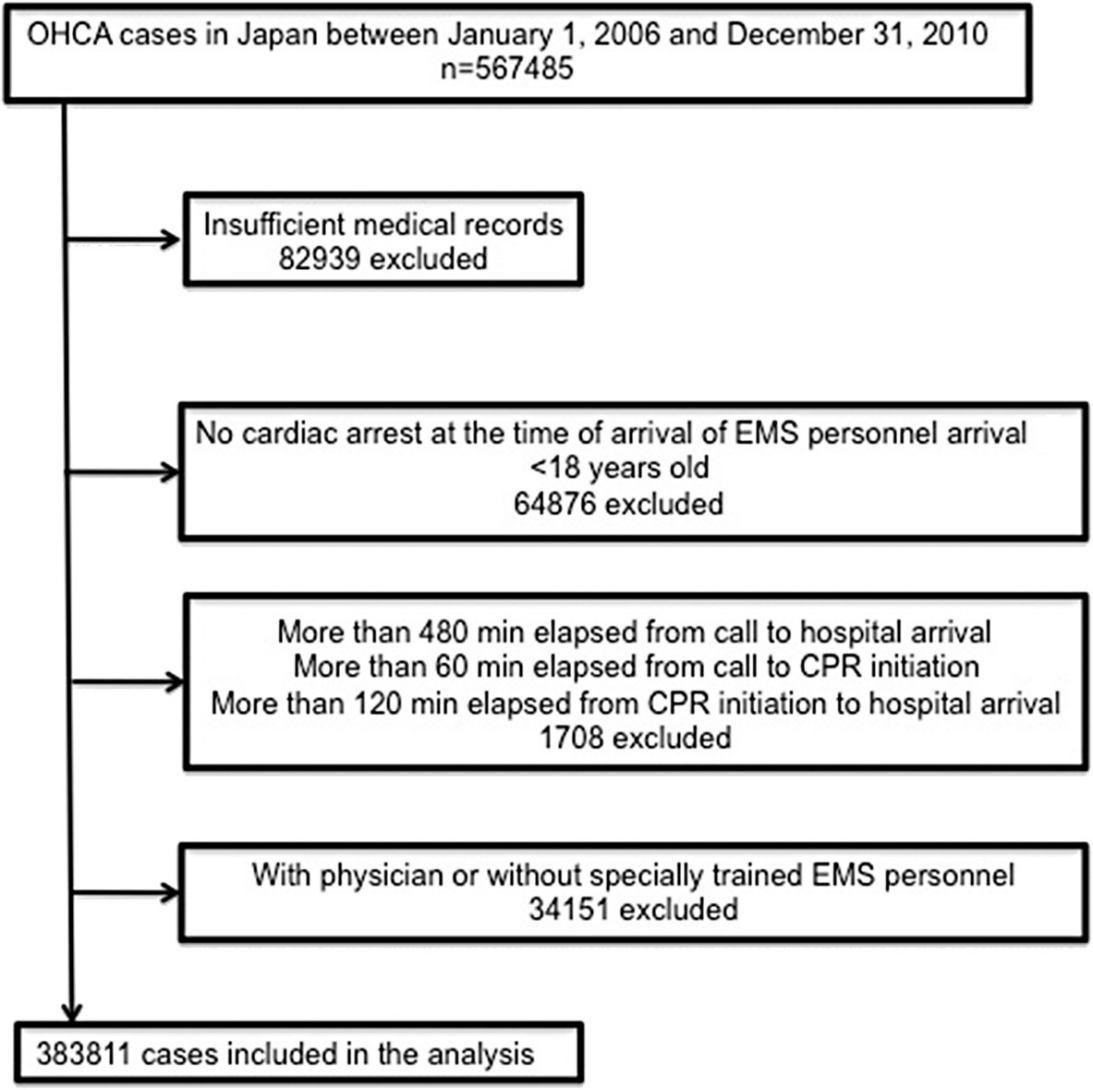
Study participant selection. *OHCA* out-of-hospital cardiac arrest, *EMS* emergency medical service

During the study period, 567,485 patients with OHCA were transferred to a hospital by EMS personnel. Of these patients, 82,939 for whom data were missing, 56,404 who had not experienced cardiac arrest or whose spontaneous circulation had been restored upon EMS personnel arrival, 8,472 who had been younger than 18 years, 130 for whom more than 480 min had elapsed from the emergency call to hospital arrival, 1,255 for whom more than 60 min had elapsed from the emergency call to CPR initiation, 323 for whom more than 120 min had elapsed from CPR initiation to hospital arrival, 12,956 who had been transferred with a physician, and 21,195 who had not been transferred with specially trained EMS personnel were excluded. After their exclusion, the 383,811 remaining patients were included in the analysis in the present study (Fig. 1).

### Patient characteristics

Table 1 shows the characteristics of the two epinephrine administration groups and Table 2 the characteristics of the four EMSs’ CPR-duration groups. As can be observed, the value of the first, second, and third quartile of EMSs’ CPR duration was 15, 20, and 27 min, respectively.

**Table 1:** Characteristics of patients with out-of-hospital cardiac arrest according to epinephrine administration (*n* = 383,811)

	Epinephrine	No Epinephrine	*P* value
	(*n* = 29067)	(*n* = 354744)	
Age, mean (SD), years	73.24 (15.3)	73.35 (16.4)	0.275
Male sex	18271 (62.9)	203925 (57.5)	<0.001
Bystander eyewitness	16081 (55.3)	121046 (34.1)	<0.001
CPR initiated by bystander	13692 (47.1)	148974 (42.0)	<0.001
Chest compression	13559 (46.9)	146701 (41.5)	<0.001
Rescue breathing	3955 (13.7)	45330 (12.9)	<0.001
Use of public-access AED	292 (1.0)	1690 (0.5)	<0.001
Intrinsic origin of cardiac arrest			
Cardiac	16652 (60.2)	183411 (53.9)	<0.001
Non cardiac	6344 (23.0)	90971 (26.7)	
Exogenous origin	4646 (16.8)	66004 (19.4)	
Primary ECG rhythm			
VF	3899 (13.4)	24064 (6.8)	<0.001
Non VF	25168 (86.6)	330680 (93.2)	
Life support by EMS personnel			
Defibrillation	5797 (20.0)	33236 (9.4)	<0.001
Advanced airway management	21866 (75.3)	160501 (45.3)	<0.001
Time, mean (SD), min			
Time from call to CPR initiation	9.37 (4.3)	9.11 (4.5)	<0.001
Time from call to arrival at the hospital	37.6 (11.9)	31.5 (11.4)	<0.001
EMSs’ CPR duration	26.5 (10.1)	21.8 (9.9)	<0.001
Outcome			
ROSC	5088 (17.5)	14349 (4.0)	<0.001
1-month survival	1448 (5.0)	13258 (3.7)	<0.001
CPC 1 or 2	339 (1.2)	5430 (1.5)	<0.001

Data are expressed as number (%), unless otherwise indicated

*CPR* cardiopulmonary resuscitation, *AED* automated external defibrillator, *VF* ventricular fibrillation, *EMS* emergency medical service, *ROSC* return of spontaneous circulation, *CPC* cerebral performance category

**Table 2:** Characteristics of patients according to the duration of EMSs’ CPR

Group	Group 1			Group 2			Group 3			Group 4		
	<15 min			15–19 min			20–26 min			≥27 min		
Characteristics	Epinephrine	No epinephrine	*P* value	Epinephrine	No epinephrine	*P* value	Epinephrine	No epinephrine	*P* value	Epinephrine	No epinephrine	*P* value
Cases	2766	90,681		5597	90,281		8179	85,490		12,525	88,292	
Age, mean (SD), years	72.91 (15.1)	72.78 (16.7)	0.669	73.61 (14.9)	73.90 (16.2)	0.192	73.75 (15.4)	73.70 (16.2)	0.784	72.81 (15.4)	73.03 (16.3)	0.159
Male sex	1,673 (60.5)	52,377 (57.8)	0.004	3409 (60.9)	50,981 (56.5)	<0.001	5103 (62.4)	48,810 (57.1)	<0.001	8086 (64.6)	51,757 (58.6)	<0.001
Bystander eyewitness	1,649 (59.6)	34,143 (37.7)	<0.001	3032 (54.2)	29,389 (32.6)	<0.001	4415 (54.0)	28,018 (32.8)	<0.001	6985 (55.8)	29,496 (33.4)	<0.001
CPR initiated by bystander (non medically trained citizen)	1,407 (50.9)	39,423 (43.5)	<0.001	2755 (49.2)	39,778 (44.1)	<0.001	3897 (47.6)	35,778 (41.9)	<0.001	5633 (45.0)	33,995 (38.5)	<0.001
Chest compression	1391 (50.5)	38,763 (42.9)	<0.001	2725 (49.0)	39,206 (43.6)	<0.001	3863 (47.5)	35,251 (41.4)	<0.001	5580 (44.8)	33,481 (38.0)	<0.001
Rescue breathing	427 (15.5)	12,185 (13.5)	0.003	776 (14.0)	11,957 (13.3)	0.162	1112 (13.7)	10,725 (12.6)	0.005	1640 (13.2)	10,463 (11.9)	<0.001
Use of public-access AED	31 (1.1)	632 (0.7)	0.012	58 (1.0)	375 (0.4)	<0.001	82 (1.0)	333 (0.4)	<0.001	121 (1.0)	350 (0.4)	<0.001
Origin of cardiac arrest												
Cardiac	1407 (54.3)	45,556 (52.9)	0.065	3110 (58.9)	46,811 (54.3)	<0.001	4681 (60.4)	44,692 (54.4)	<0.001	7454 (62.0)	46,352 (53.9)	<0.001
Not cardiac	694 (26.8)	22,557 (26.2)		1216 (23.0)	22,906 (26.6)		1763 (22.8)	22,020 (26.8)		2671 (22.2)	23,488 (27.3)	
Exogenous origin	492 (19.0)	17,958 (20.9)		954 (18.1)	16,538 (19.2)		1305 (16.8)	15,380 (18.7)		1895 (15.8)	16,128 (18.8)	
Primary ECG rhythm												
VF	467 (16.9)	9515 (10.5)	<0.001	747 (13.3)	5223 (5.8)	<0.001	1029 (12.6)	4743 (5.5)	<0.001	1656 (13.2)	4583 (5.2)	<0.001
non VF	2299 (83.1)	81,166 (89.5)		4850 (86.7)	85,058 (94.2)		7150 (87.4)	80,747 (94.5)		10,869 (86.8)	83,709 (94.8)	
Life support by EMS personnel												
Defibrillation	562 (20.3)	11,192 (12.4)	<0.001	1011 (18.1)	7443 (8.3)	<0.001	1494 (18.3)	7144 (8.4)	<0.001	2730 (21.8)	7457 (8.5)	<0.001
Advanced airway management	1822 (65.9)	26,738 (29.5)	<0.001	3981 (71.2)	40,621 (45.0)	<0.001	6258 (76.6)	44,293 (51.9)	<0.001	9805 (78.4)	48,849 (55.4)	<0.001
Time, mean (SD), min												
Time from call to CPR initiation	8.63 (3.7)	8.45 (4.5)	0.034	8.58 (3.5)	8.70 (3.8)	0.02	8.99 (3.6)	9.14 (4.0)	0.001	10.13 (5.0)	10.20 (5.4)	0.188
Time from call to arrival at hospital	29.00 (10.8)	21.95 (7.1)	<0.001	29.26 (7.4)	26.85 (4.4)	<0.001	33.51 (5.8)	32.47 (4.6)	<0.001	45.83 (11.5)	44.98 (11.5)	<0.001
EMSs’ CPR duration	12.61 (2.3)	11.51 (3.3)	<0.001	18.25 (1.3)	18.01 (1.4)	<0.001	23.42 (1.7)	23.26 (1.7)	<0.001	35.17 (9.0)	34.74 (9.3)	<0.001
Outcome												
ROSC	1561 (56.4)	11,860 (13.1)	<0.001	1,413 (25.2)	1430 (1.6)	<0.001	1211 (14.8)	665 (0.8)	<0.001	903 (7.2)	394 (0.4)	<0.001
1-month survival	503 (18.2)	8867 (9.8)	<0.001	418 (7.5)	2181 (2.4)	<0.001	297 (3.6)	1,284 (1.5)	<0.001	230 (1.8)	926 (1.0)	<0.001
CPC 1 or 2	136 (4.9)	4522 (5.0)	0.924	92 (1.6)	484 (0.5)	<0.001	59 (0.7)	239 (0.3)	<0.001	52 (0.4)	185 (0.2)	<0.001

The data are expressed as number (%), unless otherwise indicated

*EMS* emergency medical service, *CPR* cardiopulmonary resuscitation, *AED* automated external defibrillator, *VF* ventricular fibrillation, *EMS* emergency medical service, *ROSC* return of spontaneous circulation, *CPC* cerebral performance category

### Patient outcomes

Table 3 shows the ORs for the outcomes of the four EMSs’ CPR-duration groups adjusted for all the covariates listed in Table 1. In all groups, the epinephrine administration increased the rate of ROSC approximately tenfold. However, the beneficial effects of epinephrine administration on 1-month survival disappeared in group 4, and the beneficial effects of epinephrine administration on neurological outcomes were observed only in group 2 (OR, 1.327, 95 % CI, 1.017–1.733 *P* = 0.037).

**Table 3:** Adjusted odds ratios of epinephrine use for outcomes in the four EMSs’ CPR-duration groups

Group	Group 1	Group 2	Group 3	Group 4
	<15 min	15–19 min	20–26 min	≥27 min
ROSC				
OR	10.457	10.998	10.635	9.174
95 % CI	6.573–16.634	7.797–15.515	7.349–15.395	6.074–13.856
*P* value	<0.001	<0.001	<0.001	<0.001
1-month survival				
OR	1.425	1.538	1.386	1.124
95 % CI	1.254–1.619	1.347–1.755	1.200–1.602	0.959–1.318
*P* value	<0.001	<0.001	<0.001	0.15
CPC 1 or 2				
OR	0.967	1.327	0.967	1.03
95 % CI	0.774–1.027	1.017–1.733	0.774–1.207	0.728–1.458
*P* value	0.766	0.037	0.766	0.866

*EMS* emergency medical service, *CPR* cardiopulmonary resuscitation, *ROSC* return of spontaneous circulation, *OR* odds ratio, *CPC* cerebral performance category

## Discussion

Analysis of the data contained in the All-Japan UT stein registry database indicates that epinephrine administration to OHCA patients had improved the ROSC frequency by nearly tenfold as well as the 1-month survival of all patients except those on whom EMSs’ CPR had been performed for more than 26 min (group 4) and the neurological outcomes of patients on whom EMSs’ CPR had been performed between 15 and 19 min (group 2).

The finding that epinephrine had not improved the 1-month survival of group 4 indicates that epinephrine administration may not be effective for patients who undergo EMSs’ CPR of a relatively long duration. The findings that resuscitation with epinephrine administration yielded favorable neurological outcomes in only group 2 indicate that epinephrine administration is associated with improvement in the neurological outcomes of only OHCA patients on whom EMSs’ CPR is performed between 15 and 19 min. We consider in group 1 that epinephrine administration is effective like chest compression and defibrillation et al., and in group 3 and 4, epinephrine administration would not be effective because of long EMSs’ CPR duration.

Using the same database used in this study to conduct propensity score matching, Hagihara et al. [9] found that prehospital epinephrine administration to OHCA patients is significantly associated with an increased rate of ROSC before hospital arrival but decreased rates of survival and good neurological outcome 1 month after the event. However, several controversial aspects of Hagihara et al.’s study should be considered when assessing their findings in relation to the findings of the current study, foremost among which is the fact that Hagihara et al. did not consider the duration of CPR. As it is conceivable that the neurological outcomes of patients on whom EMSs’ CPR of long duration had been performed are worse than those of patients on whom EMSs’ CPR of short duration had been performed, the former were more likely to have undergone epinephrine administration by EMS staff and experience less favorable neurological outcome. Evidence of the significance of EMSs’ CPR duration was obtained in the current study, specifically the finding that the neurological outcomes of patients in group 2 had been improved by epinephrine administration. Another controversial aspect of Hagihara et al.’s study was its examination of propensity variables that we argue should not have been included in the analysis. One such variable was the presence of specially trained EMS personnel in the ambulance despite the fact that EMS personnel who are not specially trained are not permitted to administer epinephrine in Japan. We therefore argue that the researchers should have excluded patients who had been treated by EMS personnel who had not been specially trained. Another variable that we argue should not have been included was the insertion of an intravenous line, as EMS personnel are not permitted to administer epinephrine without inserting a venous line. Furthermore our study period was different from Hagihara et al.’s study period; in April 2005, prehospital epinephrine administration was introduced in Japan, so utilization in the present study is higher than their study. This difference might affect the present findings.

Using the same database as that used by Hagihara et al. to conduct time-dependent propensity score matching, Nakahara et al. [17] recently found that prehospital epinephrine administration by EMS personnel improved 1-month survival but only minimally improved the absolute increase in neurologically intact survival. However, as had Hagihara et al., they did not consider the impact of EMSs’ CPR duration. Based on the results of a recent randomized control trial that compared the effects of epinephrine and a placebo in OHCA patients, Jacobs et al. [18] and Olasveengen et al. [8] reported that epinephrine administration increases the frequency of ROSC in OHCA patients but does not improve survival in the period prior to hospital discharge. However, like the studies preceding them, neither study considered EMSs’ CPR duration when comparing the outcomes. In the present study, most of the good neurological outcomes were experienced by patients in group 1 who had not been administered with epinephrine (*n* = 4.522). If the OHCA patients had been classified by EMSs’ CPR duration, the good neurological outcome of patients in group 2 who had been administered with epinephrine (*n* = 92) might have been suppressed by the large number of good neurological outcomes experienced by patients in group 1 who had not been administered with epinephrine. Therefore, randomized controlled trials by Jacobs et al. and Olasveengen et al. might have failed to describe the effects of prehospital epinephrine administration on OHCA.

The decreased favorable neurological outcomes associated with epinephrine use can be explained by epinephrine-induced myocardial dysfunction [19, 20], ventricular arrhythmia during the period after resuscitation [21], or disturbed cerebral microcirculation following cardiac arrest [22]. Another suspected factor is provision of EMSs’ CPR of inadequate quality with epinephrine administration, as the latter requires performance of procedures that are time consuming, such as establishing intravenous access, and preparing and administering drugs and saline, and could thus potentially decrease focus on providing quality EMSs’ CPR [23–25]. Although Olasveengen et al. [8] reported that epinephrine administration is not associated with poor quality EMSs’ CPR, the effects of epinephrine administration may be minimal or nil [26] in patients who receive inadequate EMSs’ CPR quality. Also, the present study showed that prehospital epinephrine administration did not decrease favorable neurological outcomes of all groups.

Although the results of the present study might provide evidence of an association between epinephrine administration and favorable outcomes in OHCA patients, they should be reviewed with consideration of the study’s limitations. First, the quartile classification of EMSs’ CPR duration might overestimate possible association, inflate type 1 risk, and overestimate odds ratio. Second, it was not possible to determine the timing of epinephrine administration. Therefore, it was difficult to determine whether the duration of EMSs’ CPR had been determined prior to epinephrine administration, although it is equally possible that absence of response to epinephrine results in longer EMSs’ CPR duration and that EMSs’ CPR duration is an outcome rather than a predictor. Third, influence of time interval of cardiac arrest is very important for neurological outcome, although we cannot know this interval from the present study for non-witness OHCA patients who were included. Fourth, from the database we cannot know the processes and the treatments after hospital admission. Data regarding analysis of the effects of various procedures performed in the hospital, such as administration of epinephrine or other vasopressor agents (e.g., vasopressin), induction of hypothermia [27, 28], and mechanical chest compression [29], were not available. Nevertheless, it was possible that the patients who had neither experienced ROSC nor been treated with epinephrine may have been administered with epinephrine by physicians after arriving at the hospital, as well as that patients who had experienced ROSC and been administered with epinephrine had received treatment with induced hypothermia in the hospital. Fifth, the neurological outcomes were assessed by each hospital in a non-standardized manner, making comparison of the outcomes at the different hospitals difficult. Finally the cerebral performance category (CPC) scale used in the study provides limited assessment of reliability and validity [30–32]. Sixth, epinephrine administration was discretionally charged to personnel at the scene and region. Consequently epinephrine was less frequently given in all subgroup patients according to the duration of the EMSs’ CPR. This might produce bias by unidentified confounding factors. Seventh, the present study enrolled OHCA patients with non-cardiac cause or non-ventricular fibrillation (VF) rhythm. We expect that for these cases the result might be different because epinephrine administration to these patients is not recognized as a standard.

## Conclusions

The results of this study indicate that while epinephrine administration is associated with an increase of the survival of all OHCA patients, it is associated with improvement in the neurological outcomes of only OHCA patients on whom EMSs’ CPR is performed between 15 and 19 min. Consideration of these findings leads us to conclude that administration of epinephrine to OHCA patients might be supported as a means of yielding favorable outcomes. As it is difficult to discuss the effect of epinephrine administration on OHCA patients using observational data, a large, randomized controlled trial of epinephrine administration is now required.
